# The reliability of the College of Intensive Care Medicine of Australia and New Zealand “Hot Case” examination

**DOI:** 10.1186/s12909-024-05516-w

**Published:** 2024-05-11

**Authors:** Kenneth R. Hoffman, David Swanson, Stuart Lane, Chris Nickson, Paul Brand, Anna T. Ryan

**Affiliations:** 1 Intensive Care Unit, The Alfred Hospital, Melbourne, Australia; 2 Department of Epidemiology and Preventative Medicine, School of Public Health, Monash University, Melbourne, Australia; 3 Department of Medical Education, Melbourne Medical School, University of Melbourne, Melbourne, Australia; 4 Sydney Medical School, The University of Sydney, Sydney, Australia; 5 College of Intensive Care Medicine of Australia and New Zealand, Melbourne, Australia

**Keywords:** Intensive care, Examination, Credentialling, Reliability, Generalisability theory

## Abstract

**Background:**

High stakes examinations used to credential trainees for independent specialist practice should be evaluated periodically to ensure defensible decisions are made. This study aims to quantify the College of Intensive Care Medicine of Australia and New Zealand (CICM) Hot Case reliability coefficient and evaluate contributions to variance from candidates, cases and examiners.

**Methods:**

This retrospective, de-identified analysis of CICM examination data used descriptive statistics and generalisability theory to evaluate the reliability of the Hot Case examination component. Decision studies were used to project generalisability coefficients for alternate examination designs.

**Results:**

Examination results from 2019 to 2022 included 592 Hot Cases, totalling 1184 individual examiner scores. The mean examiner Hot Case score was 5.17 (standard deviation 1.65). The correlation between candidates’ two Hot Case scores was low (0.30). The overall reliability coefficient for the Hot Case component consisting of two cases observed by two separate pairs of examiners was 0.42. Sources of variance included candidate proficiency (25%), case difficulty and case specificity (63.4%), examiner stringency (3.5%) and other error (8.2%). To achieve a reliability coefficient of > 0.8 a candidate would need to perform 11 Hot Cases observed by two examiners.

**Conclusion:**

The reliability coefficient for the Hot Case component of the CICM second part examination is below the generally accepted value for a high stakes examination. Modifications to case selection and introduction of a clear scoring rubric to mitigate the effects of variation in case difficulty may be helpful. Increasing the number of cases and overall assessment time appears to be the best way to increase the overall reliability. Further research is required to assess the combined reliability of the Hot Case and viva components.

## Background

Credentialling medical specialists requires defined performance standards [1, 2] and traditionally relies upon high stakes examinations to assess trainees against those standards [3–5]. These examinations substitute for controlling quality of care by attempting to control progression through training programs for the safety of both patients and society. Specialist colleges are also expected to provide transparent and fair assessment processes, to ensure defensible decisions are made regarding trainee progression and specialist credentialling [6].

The College of Intensive Care Medicine of Australia and New Zealand (CICM) second part examination was introduced in 1979 and has undergone many revisions [3]. It has two components: a written examination and, if completed successfully, an oral examination. The oral examination includes an eight-station viva assessment and two clinical “Hot Case” assessments. This Hot Case component targets the highest level of assessment on Miller’s Pyramid [7], ‘Does’, requiring candidates to be assessed in the workplace performing real-world tasks. Of the candidates who have passed the written examination successfully, only 35% pass both Hot Cases [8]. It is therefore important to evaluate both the validity of inferences from this examination component and the reliability or reproducibility of the results [9].

Reliability describes the degree to which variation in scores reflects true variability in candidates’ proficiency, rather than measurement error. This is dependent on the task, examiner stringency and assessment context [10]. Reliability can be quantified using the reliability coefficient, with 0 representing a completely unreliable assessment and 1 representing a completely reliable assessment. The minimum standard generally considered acceptable for high stakes medical examinations is a reliability coefficient greater than 0.8 [11–14].

Generalisability theory (G-theory) provides the statistical basis for combining multiple sources of variance into a single analysis [15]. This enables the calculation of an overall reliability coefficient and calculation of the contribution from candidates, cases and examiners to examination reliability. G-theory also provides the basis for conducting decision studies (D-studies) that statistically project reliability based on alternate assessment designs.

To date, no information on the reliability of the CICM second part examination has been published. Given the implications of incorrect credentialling decisions for trainees, patients and society, the Hot Case reliability coefficient should be quantified.

## Methods

### Examination format

The second part examination prior to COVID-19 was held twice yearly with candidates invited to the oral component in a single Australian city. Trainees complete two Hot Cases within metropolitan intensive care units (ICU) with 20 min allocated for each: 10 min to examine an ICU patient, followed by 10 min with paired examiners to present their findings and answer questions regarding investigations and clinical management.

Format changes occurred during the COVID-19 pandemic. The first oral examination was cancelled in 2020, with trainees deferring to the second sitting. Additionally, travel restrictions meant candidates sat the Hot Case component in their home city with local examiners from the second sitting in 2020 to the second sitting in 2021. From 2022 onwards, the oral examination has been held in Sydney, Melbourne, or both.

Hot Cases are marked out of 10 by two CICM examiners using a rating scale that scores candidates based on how comfortable examiners would be supervising them. An acceptable pass standard (5/10) indicates an examiner is comfortable to leave the candidate in charge of the ICU with minimal supervision. There is no specific scoring rubric, although examiner pairs cooperatively determine clinical signs that should be identified, nominate investigations and imaging to show a candidate, and specify discussion questions. Expected levels of knowledge, interpretation and clinical management are defined prospectively. An automatic fail for the entire oral examination is triggered if candidates fail both Hot Cases and obtain a Hot Case component mark < 40% of the possible marks.

### Examiner calibration

Examiners undergo calibration training prior to the examination. They independently score the candidate, then discuss their individual scores and rationale. Examiners can then amend their score before recording final scores in the examination database. Each Hot Case is marked by separate pairs of examiners, to prevent bias from a candidates first case performance influencing their second case score. Following the examination, results are presented to the whole examiner cohort for further discussion and explanation.

### Data collection

The CICM provided access to their examination database from the second sitting of 2012 (2012-2) through to the first sitting of 2022 (2022-1). For each de-identified candidate, the written mark, overall Hot Case mark, viva mark, and overall examination mark were obtained. The Hot Case specific data included the cases used, examiners present and individual examiner marks, with a total of four scores per candidate (two examiner scores for each Hot Case).

Analysis was restricted to 2019-1 to 2022-1 due to data recording inconsistency providing insufficient data for G-theory analysis. Additionally, changes occurred from 2019-1 with the introduction of the Angoff standard setting method [16, 17] for the written examination. This altered final score calculation with the written examination functioning as a barrier examination, although the written score no longer contributes to the final examination score. Candidates were included if they sat the oral examination for the first time in 2019 or later and, if they failed, subsequent attempts were recorded.

### Statistical analysis

Statistical analysis used Microsoft Excel and SPSS. Continuous examination scores were summarised using mean and standard deviation. Categorical variables were reported as counts and percentages. Frequency distributions (histograms) were used to graph overall examination component results. A p-value of < 0.05 indicated statistical significance. Comparisons of examiner marks and relationships between examination components were analysed with Pearson’s correlation coefficient and visually represented with scatterplots.

**Fig. 1:**
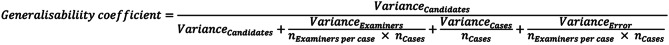
Generalisability coefficient equation

G-theory analysis was used to calculate an overall reliability coefficient for the Hot Case examination, and the factors contributing to variance. As examiners observed multiple candidates and candidates performed multiple Hot Cases, the design was partially crossed. However, as the case identification numbers used in the examination were recorded variably, the initial design was modified to treat cases as nested within candidates for the analysis. The variance factors being analysed included candidate proficiency, examiner stringency, case to case performance variability (case specificity) and other unspecified measurement error. These were reported with variance components, square roots of variance components and percentage of total variance. G-theory was used to conduct D-studies exploring the impact of alternate assessment designs on overall generalisability coefficients and associated standard errors of measurement. The D-study calculated the generalisability coefficient based on the equation listed in Fig. 1.

## Results

**Fig. 2:**
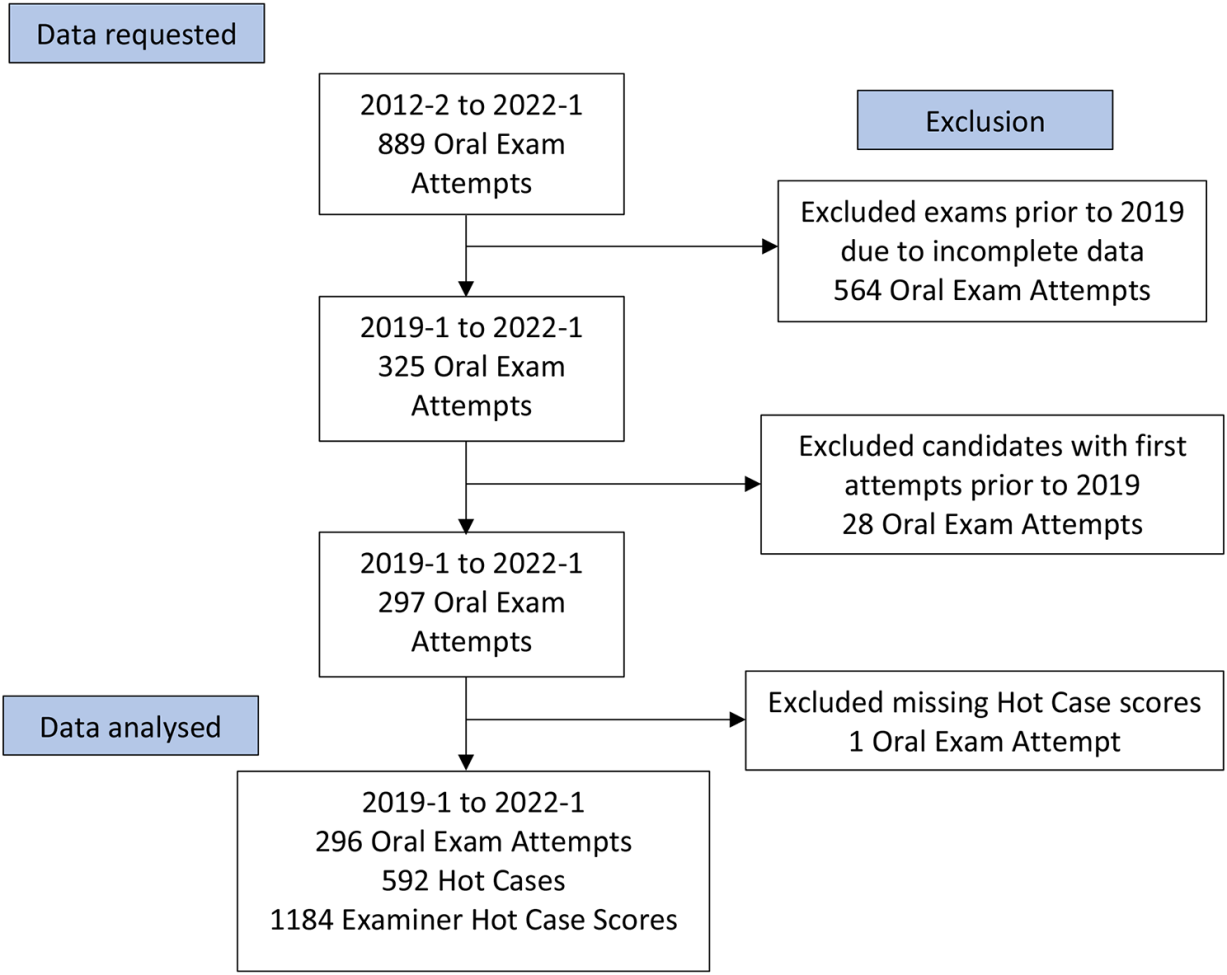
CONSORT style diagram demonstrating the sample size from data request through to the sample available for analysis

Overall, there were 889 candidate oral examination attempts from 2012-2 to 2022-1. After exclusion of candidate oral examination attempts prior to the 2019-1 sitting, exclusion of candidates with first attempts prior to 2019-1 and exclusion of one candidate with missing Hot Case scores, there were 296 candidate oral examination attempts analysed. This included 166 first attempts, 100 s and 30 third attempts. This resulted in 592 Hot Case results and 1184 individual examiner Hot Case scores. The recruitment, exclusion and analysis of the sample are presented in Fig. 2.

**Fig. 3:**
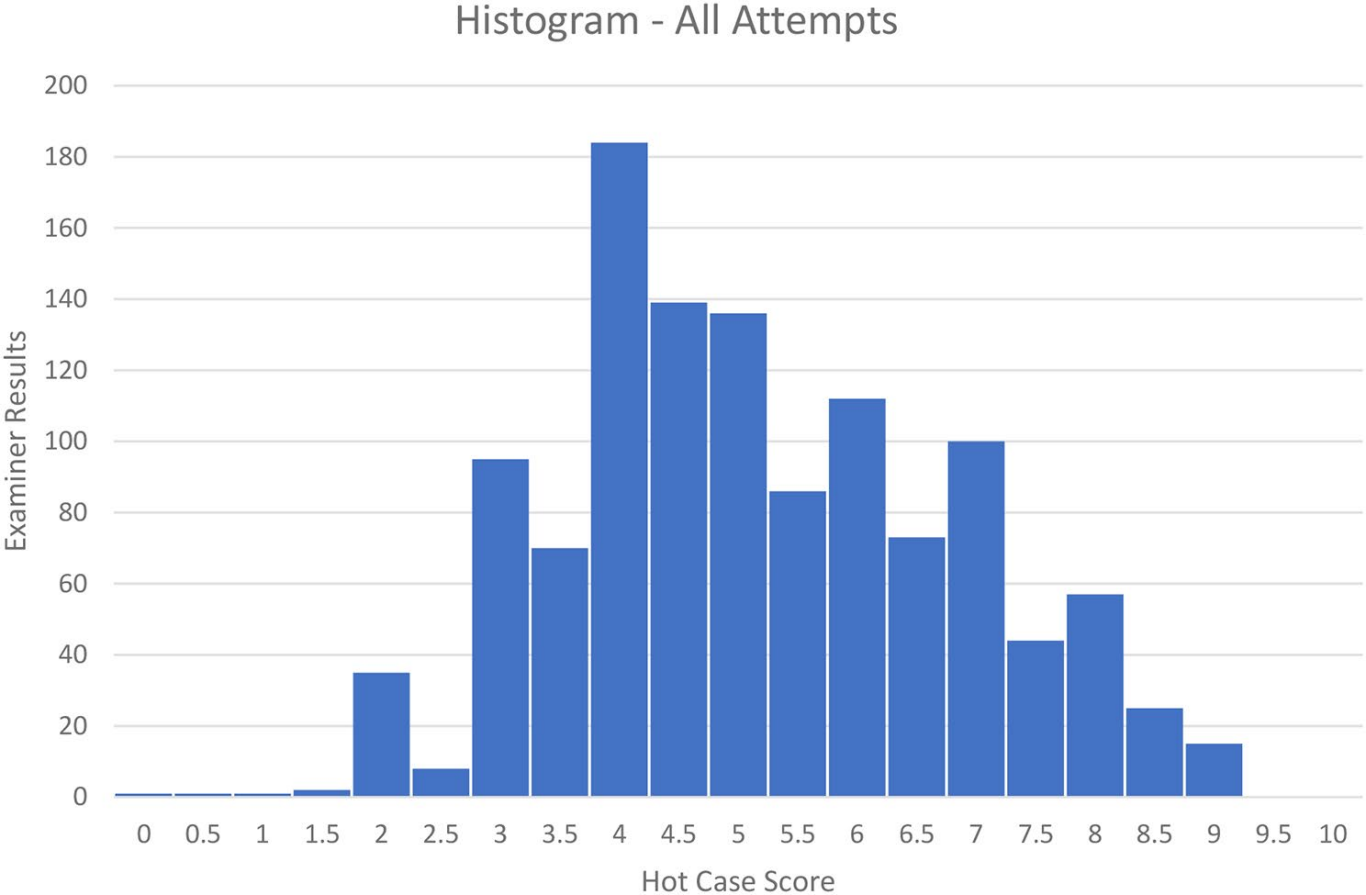
Histogram showing individual examiner Hot Case scores for all attempts

The mean and standard deviation of individual examiner Hot Case scores from all examiners was 5.17 and 1.65 respectively. Of the 1184 Hot Case individual examiner scores, 645 (54.5%) achieved a score 5 or greater, and 539 (45.5%) scored less than 5. The distribution of individual examiner Hot Case scores is presented in Fig. 3. First attempt candidates scored higher than those repeating (5.25 (SD1.63) vs. 4.89 (SD1.66) *p* = < 0.01).

**Fig. 4:**
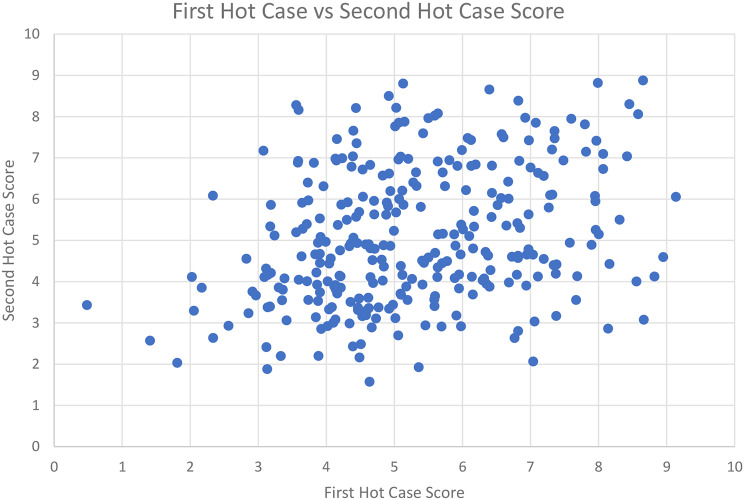
The correlation between each candidate’s first and second Hot Case scores. A jitter function was applied to spread overlying points

Scores on each Hot Case are calculated as the mean of the two individual examiner Hot Case scores. Overall, 312 of 592 Hot Cases were passed (52.7%). The correlation coefficient between candidates first and second Hot Cases was low at 0.30 (Fig. 4).

**Fig. 5:**
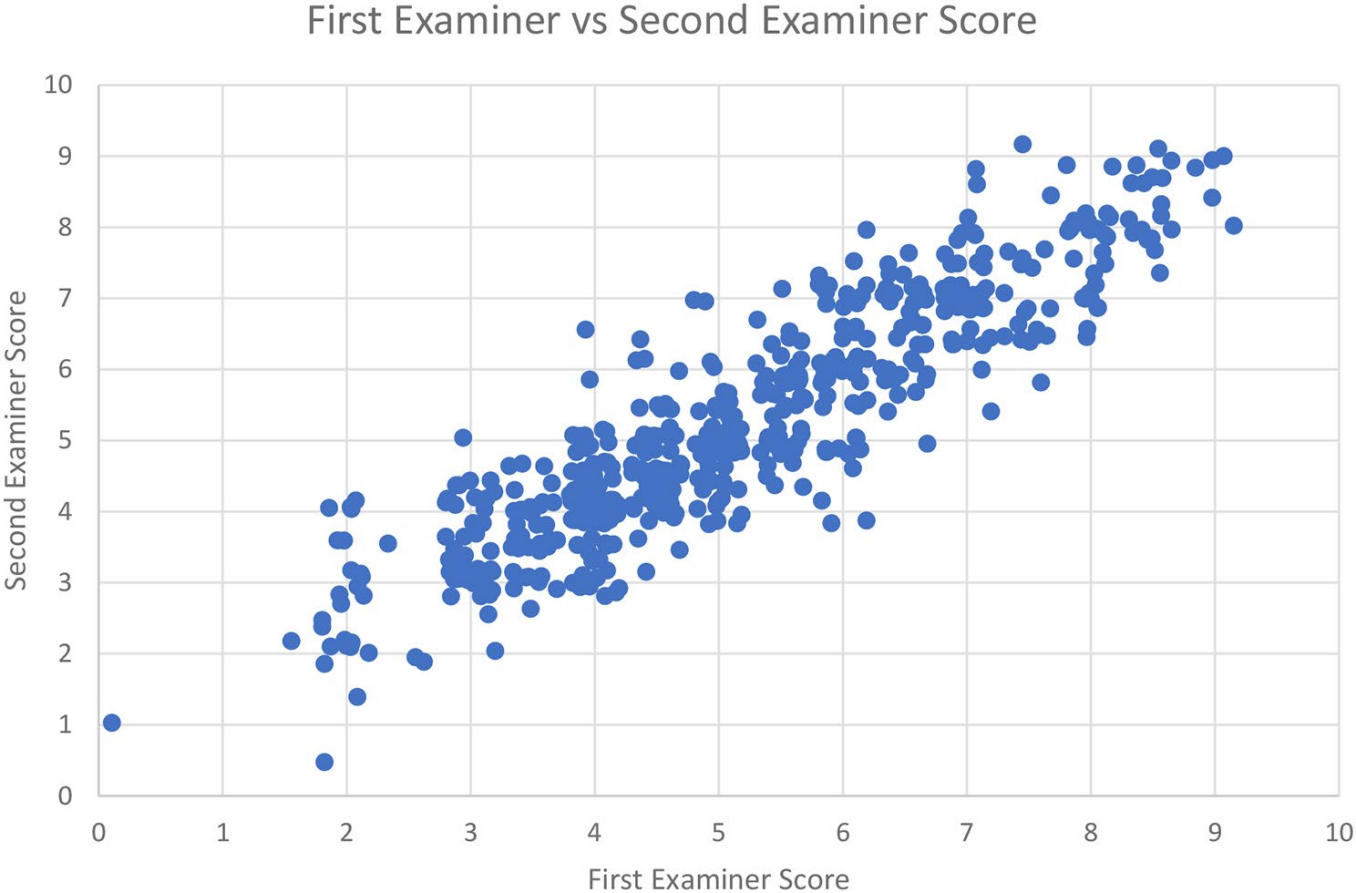
Comparison between Hot Case scores from the first and second examiners. A jitter function was applied to spread overlying points

The correlation coefficient between examiners observing the same case (inter-rater agreement) was high at 0.91 (Fig. 5).

**Table 1:** Sources of variance

Source of variance	Variance component (VC)	Square root of VC	VC as % of total variance	Interpretation				
Candidates	0.6512	0.81	25.0	“True” variation in candidate proficiency if no measurement error were present				
Examiners	0.0910	0.30	3.5	“True” variation in examiner stringency if no measurement error were present (hawk/dove differences)				
Cases(Candidates)	1.6534	1.29	63.4	“True” variation in case difficulty and candidate performance (case specificity) if no measurement error were present				
Other Error	0.2131	0.46	8.2	Other sources of measurement error				

Sources of variance for Hot Case scores

The summary of sources of variance for individual examiner Hot Case scores is presented in Table 1.

The overall generalisability coefficient of the Hot Case component including two separate cases observed by two examiners each was 0.42.

**Fig. 6:**
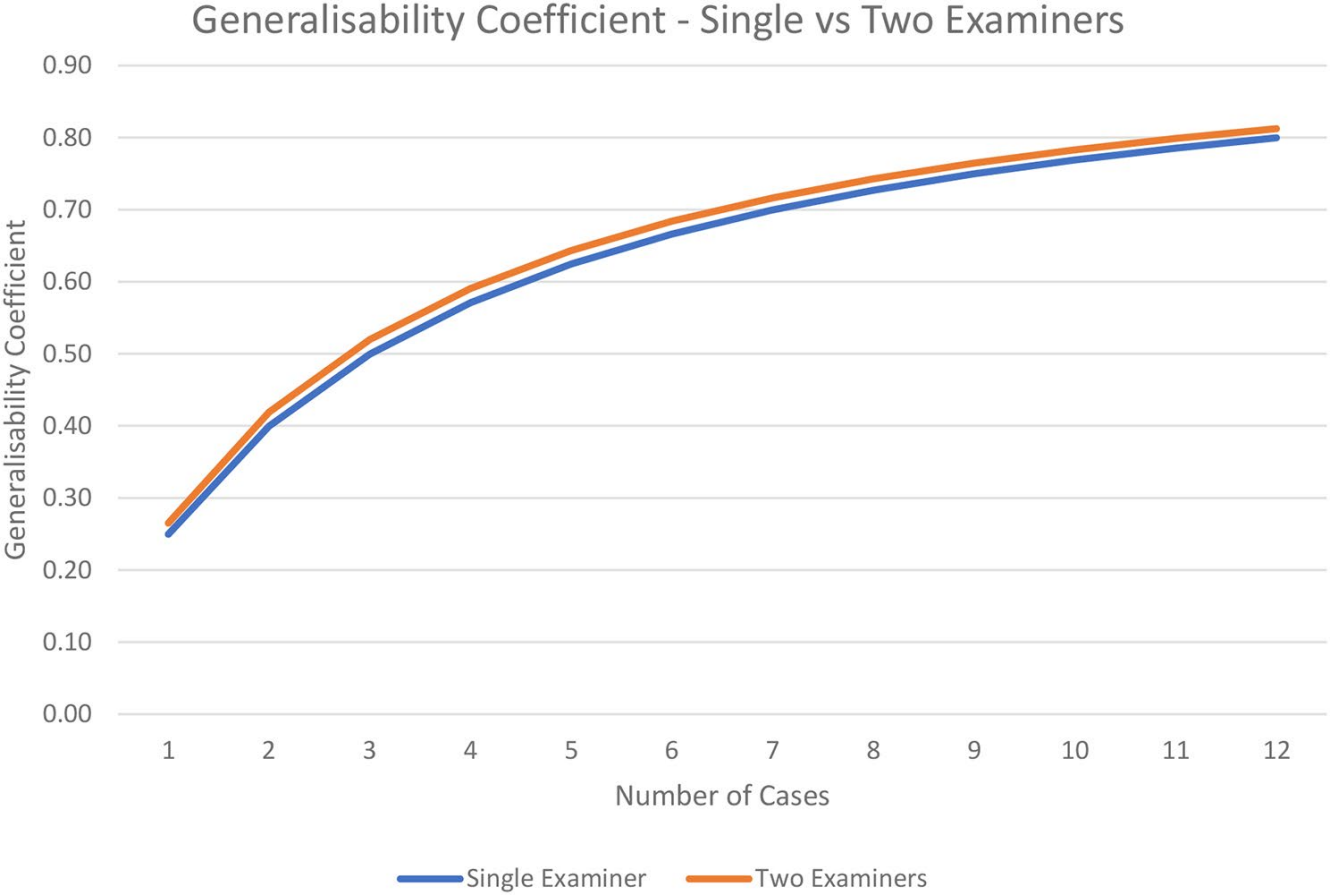
Generalisability coefficients with a variable number of cases comparing examination designs with one and two examiners observing each case

The results for the D-studies are presented in Table 2. To achieve a generalisability coefficient of 0.8 or greater, 11 Hot Cases with two examiners would be needed. A graph comparing the generalisability coefficients for one and two examiners is presented in Fig. 6.

**Table 2:** Projected generalisability coefficients

Cases	Single examiner	Two examiners
1	0.25	0.27
2	0.40	0.42
3	0.50	0.52
4	0.57	0.59
5	0.62	0.64
6	0.67	0.68
7	0.70	0.72
8	0.73	0.74
9	0.75	0.76
10	0.77	0.78
11	0.79	0.80
12	0.80	0.81

Projected generalisability coefficients for various combinations of test lengths with single vs. two examiner designs

## Discussion

The current examination format with two Hot Cases observed by two examiners has a reliability coefficient of 0.42. To achieve the widely accepted standard for high stakes examinations of a reliability coefficient of > 0.8 requires each candidate to sit 11 Hot Cases with two examiners.

These results are similar to The Royal Australasian College of Physicians (RACP) 60-minute long case examination observed by two examiners which has a reliability coefficient of 0.38 [18]. When the assessment time is lengthened with two long cases and four short cases, the RACP achieved a reliability coefficient of 0.71. The RACP continues to use long case examinations, as they are valued by examiners and trainees as an authentic measure of competence with an educational impact from examination preparation [18]. Educational impact is commonly cited as a reason to retain clinical examinations [4, 19, 20].

G-theory analysis demonstrates that examiners appear well calibrated, as examiner variance was responsible for only 3.5% of overall variance in Hot Case scores. Therefore, adding additional examiners would not substantially improve reliability. However, this conclusion may be affected by the extent of discussion between the examiners prior to recording their amended final scores. If discussion influences the opinions of an examiner strongly, it is likely there will be higher correlation between examiner scores. To evaluate this effect, independent examiner scores would need to be recorded prior to discussion, with clear guidelines around acceptable amendments to scores.

The finding that the majority of Hot Case variance (63.4%) arises from case variation is consistent with anecdotal reports from examination candidates who describe case difficulty as a “lucky dip”. This finding is consistent with the poor correlation (0.30) between candidates’ first and second Hot Cases. Whilst examiners preview the Hot Case patient, there is no formal method of quantifying and adjusting for the difficulty of each case. According to Kane’s Validity Framework [21], it is difficult to argue that the assessment is valid if the initial scoring and subsequent generalisation of those scores are based more on case specificity than candidate proficiency, particularly when the implications of the results are significant for candidates and patient safety. The CICM has introduced the Angoff method [16] for the written examination to account for variation in question difficulty and an appropriate standard setting method for the Hot Case component may mitigate this degree of case variability to some extent. The CICM has avoided the use of norm referenced assessments where candidates are compared with their peers so that all candidates deemed competent are eligible to pass. This is appropriate given the low number of candidates in each sitting, the low number of candidates taken to each case and high variability in case difficulty.

Case specificity is the concept that candidate performance is dependent on the case used and is a major issue in specialist credentialling examinations [4]. Problem solving ability and clinical reasoning are based on prior clinical experience, areas of particular interest and background knowledge. Candidate performance may be highly case specific, meaning limited numbers of examination cases have detrimental effects on reliability [4, 5, 22]. In the literature, increasing case numbers or overall assessment time is commonly proposed as a method of obtaining more generalisable results [6, 18, 23, 24]. However, having a candidate pass overall, but clearly fail a component of a credentialling examination may be difficult to justify as defensible from the perspective of patient safety and societal obligations.

The individual examiner Hot Case scores (5.17, SD 1.65) are close to the 50% pass fail boundary. This makes examiners’ decision making difficult, with potentially small differences in performance determining a pass or fail. This is demonstrated in the histogram in Fig. 3, with a large proportion of trainees scoring a 4.5 or 5, the junction between a pass and a fail. This dichotomisation should be supported by a clear rubric defining what constitutes a minimally competent performance. This will also give candidates clearer performance expectations and may mitigate variability due to case difficulty and specificity by defining expected competencies which are independent of the case difficulty.

Assessing the quality of future care using examination performance as a substitute marker of competence has limitations [11]. There are concerns from a validity point of view regarding decision making based on short periods of assessment [6, 9, 10, 18, 25]. As such, credentialling examinations should focus on identifying low end outliers, a possible true risk to patients and society without further training. Rather than failing candidates with a borderline performance, the focus should be on increasing the sample size to guide decision making. Additional Hot Cases for those with a borderline performance on the oral examination is a possible solution, to increase the reliability for defensible decision making. Summative Hot Cases performed during the training program, but not at the time of the final examination, is another option to increase available data through a transition to a programmatic style of longitudinal assessment.

Restricting the analysis for candidates who sat the written from the 2019-1 sitting onwards was necessitated by the quality of the available dataset. This aided analysis as the Angoff method was introduced for the written paper in 2019 [17] with the written score no longer counting toward the overall examination score. Candidates are now considered to have passed or failed the written, and then to pass the oral examination they require > 50% from the Hot Case component (worth 30 marks) and viva component (worth 40 marks) combined. This results in a higher benchmark to pass the examination overall, as previously a strong written mark could contribute to an overall pass despite a weaker oral performance.

This research fills a gap in the current understanding of credentialling intensive care physicians. However, it should be taken in context of the overall assessment process. If high stakes assessment requires a reliability coefficient of > 0.8, this value should be the benchmark for the combined oral examination including the Hot Cases and viva component. Further research is required to assess how the Hot Case component and the viva component interact to form the overall reliability of the oral examination.

The strengths of this study include the originality, the predefined statistical plan, the large cohort and the collaboration with the CICM to provide previously unexamined data for an independent analysis. Additionally, the use of descriptive statistics, G-theory analysis and D-studies provides a comprehensive picture of the Hot Case examination reliability in its current format.

Study limitations include dataset consistency issues that restricted the study period, the focus specifically on the Hot Case component without an in-depth analysis of the other components of the examination, the focus on traditional psychometric evaluation and the potential overestimation of examiner calibration due to revision of examiner scores after discussion. Evaluating examination performance without external measures of candidate ability is a research design that focuses on the examination itself. Assessment research is often not truly focussed on candidate competence as this is very difficult to study, so it inevitably evaluates the process rather than the product. As such, identifying poor reliability as a weakness of the Hot Case examination does not detract from potential validity in the overall examination process.

Several implications and unanswered questions remain. Firstly, examiners appear well calibrated, but discussion and score amendment may be significant. Secondly, with additional examiner time, reliability could be increased by challenging candidates with borderline results with additional cases upon which decisions are made. Thirdly, this research highlights the importance of a scoring rubric and robust processes for data capture. Finally, further research is required to assess how the Hot Case and viva examination interact to test the overall reliability of the oral examination. This should be supported by research aiming to assess the validity of the Hot Case as a method of evaluating clinical competence by comparing it with other forms of assessment and workplace competency.

## Conclusion

Hot Cases have long been a method of assessment in ICU training in Australia and New Zealand, with perceived benefits from the perspective of stakeholder acceptance and educational impact. Changes to the current examination format to increase reliability would solidify its role in the credentialling process by addressing concerns within the ICU community.

The reliability of the CICM Hot Case examination is less than the generally accepted standard for a high stakes credentialling examination. Further examiner training is unlikely to improve the reliability as the examiners appear to be well calibrated. Modifications to case selection and the introduction of a clear scoring rubric to mitigate the effects of variation in case difficulty may be helpful, but are unlikely to improve reliability substantially due to case specificity. Increasing the number of cases and overall assessment time appears to be the best way to increase the overall reliability. Further research is required to assess how the Hot Case and viva results interact to quantify the reliability of the oral examination in its entirety, and to evaluate the validity of the examination format in making credentialling decisions.

## Data Availability

The datasets analysed during the current study are not publicly available, but are available from the corresponding author on reasonable request.
